# OsGA2ox5, a Gibberellin Metabolism Enzyme, Is Involved in Plant Growth, the Root Gravity Response and Salt Stress

**DOI:** 10.1371/journal.pone.0087110

**Published:** 2014-01-27

**Authors:** Chi Shan, Zhiling Mei, Jianli Duan, Haiying Chen, Huafeng Feng, Weiming Cai

**Affiliations:** 1 Institute of Plant Physiology and Ecology, Shanghai Institutes for Biological Sciences, Chinese Academy of Sciences, Shanghai, China; 2 Shanghai Key Laboratory of Functional Materials Chemistry, School of Chemistry and Molecular Engineering, East China University of Science and Technology, Shanghai, China; Wake Forest University, United States of America

## Abstract

Gibberellin (GA) 2-oxidases play an important role in the GA catabolic pathway through 2β-hydroxylation. There are two classes of GA2oxs, i.e., a larger class of C_19_-GA2oxs and a smaller class of C_20_-GA2oxs. In this study, the gene encoding a GA 2-oxidase of rice, *Oryza sativa* GA 2-oxidase 5 (*OsGA2ox5*), was cloned and characterized. BLASTP analysis showed that OsGA2ox5 belongs to the C_20_-GA2oxs subfamily, a subfamily of GA2oxs acting on C_20_-GAs (GA_12_, GA_53_). Subcellular localization of OsGA2ox5-YFP in transiently transformed onion epidermal cells revealed the presence of this protein in both of the nucleus and cytoplasm. Real-time PCR analysis, along with GUS staining, revealed that *OsGA2ox5* is expressed in the roots, culms, leaves, sheaths and panicles of rice. Rice plants overexpressing *OsGA2ox5* exhibited dominant dwarf and GA-deficient phenotypes, with shorter stems and later development of reproductive organs than the wild type. The dwarfism phenotype was partially rescued by the application of exogenous GA_3_ at a concentration of 10 µM. Ectopic expression of *OsGA2ox5* cDNA in *Arabidopsis* resulted in a similar phenotype. Real-time PCR assays revealed that both GA synthesis-related genes and GA signaling genes were expressed at higher levels in transgenic rice plants than in wild-type rice; *OsGA3ox1*, which encodes a key enzyme in the last step of the bioactive GAs synthesis pathway, was highly expressed in transgenic rice. The roots of *OsGA2ox5-ox* plants exhibited increased starch granule accumulation and gravity responses, revealing a role for GA in root starch granule development and gravity responses. Furthermore, rice and *Arabidopsis* plants overexpressing *OsGA2ox5* were more resistant to high-salinity stress than wild-type plants. These results suggest that *OsGA2ox5* plays important roles in GAs homeostasis, development, gravity responses and stress tolerance in rice.

## Introduction

Gibberellins (GA) are plant hormones that govern many aspects of plant biology, including seed germination, stem elongation, leaf expansion, flowering transition, seed development and apical dominance [1]–[7]. There are more than 100 different GAs, but most of these are precursors and degradation products [8]. Bioactive GAs in higher plants include GA_1_, GA_3_, GA_4_ and GA_7_ [8]. Plants exhibiting the typical GA-deficiency phenotype are dwarfed, with small, dark green leaves, retarded growth and late flowering [9]–[12].

The GA biosynthesis pathway has long been a subject of study, and the genes encoding the main enzymes in each step of the GA biosynthesis and catabolism pathways have been identified in *Arabidopsis thaliana* and rice *(Oryza sativa)* [8], [13], [14]. In rice, several GA-related mutants have been studied in detail [10], [15]–[17]. GAs are synthesized from trans-geranylgeranyl diphosphate (GGDP) [8], [18] in three steps. In the first step, GGDP is transformed into the tetracyclic hydrocarbon ent-kaurene via ent-copalyl diphosphate (CDP) through two types of diterpene cyclases in plastids, copalyl diphosphate synthase (CPS) and ent-kaurene synthase (KS). Next, two membrane-associated P450 monooxygenases, ent-kaurene oxidase (KO) and ent-kaurenoic acid oxidase (KAO), help convert ent-kaurene into GA_12_ in the endoplasmic reticulum. The last step of GA synthesis involves soluble 2-oxoglutarate-dependent dioxygenase (2ODDs). In this step, C_20_ is oxidized and removed, leading to the formation of C_19_-GAs such as GA_9_ and GA_20_; the formation of these C_19_ GAs is catalyzed by GA 3-oxidase in the cytosol [8], [18], [19]. The main degradation pathway for GAs is catalyzed by GA 2-oxidase (GA2ox), a 2β-hydroxylation enzyme that hydroxylates C-2 of active GAs. These GA2oxs are encoded by a small gene family that has been identified in *Arabidopsis*, spinach and rice [8], [10], [20], [21]. These GA2oxs are classified into two subgroups based on the substrates that they act on, i.e., C_19_GA2oxs and C_20_GA2oxs. C_19_GA2oxs can hydroxylate the C-2 of active C_19_-GAs (GA_1_ and GA_4_), as well as C_19_-GA precursors such as GA_9_ and GA_20_, to produce the inactive forms of GAs, i.e., GA_8_, GA_34_, GA_29_ and GA_51_. The subgroup C_20_GA2oxs only acts on C_20_-GA precursors, such as GA_12_ and GA_53_, to form GA_110_ and GA_97_ [21], [22]_,_ but not C_19_-GAs. These C_20_GA2oxs contain three unique, conserved amino acid motifs that are not present in the C_19_GA2oxs subgroup [21]. The C_20_GA2oxs includes two *Arabidopsis thaliana* proteins (AtGA2ox7 and AtGA2ox8), one soybean (*Glycine max* [L.] Merr) protein (GmGA2ox4), one spinach (*Spinacia oleracea*) protein (SoGA2ox3) and three rice (*Oryza sativa*) proteins (OsGA2ox5, OsGA2ox6 and OsGA2ox9) [21]–[24]. The physiological functions of these C_20_GA2oxs have been studied in some plant species. The overexpression of *AtGA2ox7* and *AtGA2ox8* produce a dwarf phenotype with reduced GA levels, while ectopic expression of *AtGA2ox7* and *AtGA2ox8* in transgenic tobacco (*Nicotiana tabacum*) leads to a dwarf phenotype [22]. A similar phenotype was also observed in rice overexpressing *OsGA2ox6* [25]. These results suggest that C_20_GA2oxs reduce the level of bioactive GAs in plants. Another mechanism of GA degradation has recently been reported. The rice *ELONGATED UPPERMOST INTERNODE* gene (*OsEUI*) encodes a cytochrome P450 monooxygenase that catalyzes epoxidation of the C_16, 17_ double bond, which results in decreased levels of bioactive GAs [26], [27].

In this study, we report the functional characterization of the *OsGA2ox5* gene, which encodes a C_20_GA2ox enzyme in rice. Overexpression of *OsGA2ox5* in rice and *Arabidopsis* plants produced a dwarf phenotype with retarded growth; the application of exogenous GA3 rescued the GA-deficient phenotype. GA biosynthesis and GA signaling pathway genes were up-regulated in transgenic rice plants, especially *OsGA3ox1*, the last enzyme in the synthesis of bioactive GAs. We also found out that *OsGA2ox5* functions in salinity resistance and gravity responses.

## Materials and Methods

### Plant Materials and Growing Conditions

The rice cultivar Zhonghua 11 (*Oryza sativa* L. subsp. *japonica*) was used for rice transformation. Rice plants were grown in a greenhouse at 28°C. *Arabidopsis thaliana* ecotype Col-0 was used as the wild type. Plants were grown on soil or on plates containing MS medium under LD (16 h light/8 h dark) condition at 22°C.

Rice seeds were surface sterilized for 5 min with ethanol (75% v/v) and 30 min with commercially diluted (1∶3 v/v) NaOCl, followed by several rinses with sterile water. Germination was carried out for 72 h on sterile MS medium in the dark at 28°C. The plants were then grown at 28°C-day/25°C-night, under a 12-h-light/12-h-dark cycle and at a relative humidity of 50%.

### RNA Extraction and Real-time PCR Assays

Total RNA was extracted from root, stem, leaf, sheath, and panicles using the TRIzol reagent (Invitrogen) for analysis of *OsGA2ox5* mRNA expression. To analyze the transcription levels of gibberellin metabolism and signal pathway genes, 3-week-old WT and *OsGA2ox5-ox* rice seedlings were harvested and subjected to RNA extraction using the TRIzol reagent (Invitrogen). The RNA was reverse-transcribed using an oligo (dT)_ 18_ primer and AMV reverse transcriptase (Toyobo) according to the manufacture’s protocol. Real-Time PCR was performed using CFX96 (Bio-Rad, USA) and SYBR Green I (CWBIO); the Real-time PCR assays were performed in triplicate for each cDNA sample. The data were normalized using the rice marker gene *OsActin*. All primers used in this study are listed in supplemental Table S1.

### Construction of P_OsGA2ox5_:GUS Vector and Staining

The promoter region of *OsGA2ox5*, 3,500-bp upstream of ATG (*P_OsGA2ox5_*), was amplified from the rice genome by PCR using KOD polymerase (Toyobo) and inserted upstream of the *GUS* gene at the *Xba I*-*Sma I* sites of the p1300GN-GUS vector. The primers used are OsGA2ox5 gusF and OsGA2ox5 gusR (sangon) (the specific primers are listed in supplemental Table S1). The *P_OsGA2ox5_ :GUS* construct was transfected into *A. tumefaciens EHA105* by heat shock, followed by transformation of rice embryonic calli, as described previously [28]. GUS staining was used to investigate the level of *OsGA2ox5* expression in the T1 generation of *P_OsGA2ox5_ :GUS* transgenic rice. Transgenic plant samples were incubated in GUS staining solution (100 mmol/L NaH_2_PO_4_ buffer pH 7.0, 0.5% Triton X-100, 0.5 mg/ml X-Gluc and 20% methanol) overnight at 37°C. After staining, the tissues were rinsed and photographed.

### Overexpression of *OsGA2ox5* in Rice and *Arabidopsis*

The full-length CDS of *OsGA2ox5* was amplified using primers OsGA2ox5F and OsGA2ox5R (sangon) and cloned in the vector pMD-18T (TaKaRa); the sequence was confirmed by DNA sequencing. The *OsGA2ox5* CDS from the sequenced clone was removed by digestion and cloned into modified binary vector pHB [29]. The binary vector pHB-*OsGA2ox5* was transformed into *Agrobacterium* strain EHA105 and transfected into rice embryonic calli as described previously [28]; this vector was used to transform *Arabidopsis thaliana* ecotype Columbia-0 using previously described methods [30]. The transgenic plants were selected using hygromycin. The T1 plants were confirmed by PCR using the following specific primers for the *hygromycin phosphotransferase* (*HPT*) gene: 5-TGTCCTGCGGGTAAATAGC-3 and 5-TGCTCCATACAAGCCAACC-3 (AY836546). To analyze of *OsGA2ox5* gene expression level in transgenic plants, 3-week-old WT and *OsGA2ox5-ox* rice seedlings were harvested and subjected to RNA extraction using the TRIzol reagent (Invitrogen). RT-PCR was performed with oligos OsGA2ox5RTF and OsGA2ox5RTR (Table S1) using Taq DNA polymerase (TaKaRa).

### Southern Blot

Southern blot was used to analyze the transgenic plants. 20 μg of total genomic DNA from leaf tissue of transgenic plants and wild type plants was digested with appropriate restriction endonuclease *Hind III* (only one recognition site in T-DNA sequence). DNA fragments were separated by electrophoresis on a 1% (w/v) agarose gel and then transferred to a nylon membrane (Amersham Bioscience) according to standard protocols. Dig-high DNA labeling kit I (Roche) was used to label the Hygromycin DNA probes.

### Subcellular Localization of OsGA2ox5

The coding region of *OsGA2ox5* was amplified using the primer pair OsGA2ox5-pA7YFPF and OsGA2ox5-pA7YFPR (sangon) (the specific primers are listed in supplemental Table S1) and cloned into pA7-YFP [31], generating the OsGA2ox5-YFP fusion under the control of the CaMV 35S promoter. A previously study demonstrated that OsGHD7 [32] is a nuclei protein and localized in nuclei only, so we used OsGHD7 as a positive control. The *OsGHD7* coding sequence was fused in frame to the N-terminus of YFP under the control of the CaMV 35S promoter. Then, OsGA2ox5-YFP, OsGHD7-YFP fused construct and pA7-YFP vectors were used to transiently transform onion epidermal cells by particle bombardment[33] using a particle gun system (PDS-1000/He; Bio-Rad). After 24 h, the epidermal cells were examined for YFP fluorescence under a scanning confocal microscope (Zeiss LSM510; Carl Zeiss Micro-Imaging GmbH, Jena, Germany).

### Longitudinal Section Microscopic Analysis

The second leaf sheaths from 5-day-old WT and OX rice plants which were cut into 1mm width were rinsed in 75% ethanol at room temperature overnight. Then the samples were cleared for 24 h in a chloralhydrate solution (chloralhydrate-H_2_O-glycerol, 8:2:1, w:v:v) and detected in microscopic (Leica MZ95).

### Exogenous GA3 Treatment

14-day-old WT (ZH11) and *OsGA2ox5-ox* plants were incubated in 1/2 MS medium containing 1 μM GA_3_ (sangon). The seedling height (from the base to the leaf cap) was measured at day 7 after GA_3_ treatment. Plants grown in a greenhouse were sprayed with 10 µM GA_3_ three times a week.

### Root Gravitropism Analysis

The seeds were surface-sterilized and sown on half-strength MS medium containing 0.45% phytagel. Four-day-old seedlings with radicals approximately 6 cm in length were subjected to gravitropism analysis. Light-grown wild-type and *OsGA2ox5-ox* seedlings were displaced by 90° and monitored for the orientation of the primary root caps. The vertical position is represented by 90°, and the horizontal position is represented by 0°. The seedlings were reoriented by 90°, and images of the roots were captured at 0 h, 0.5 h, 1 h, 2 h, 3 h, 4 h and 5 h. The degrees of curvature were measured from the digital images using Image J software (http://rsbweb.nih.gov/ij/).

### Staining of Starch Granules

Starch granules in the root cap were visualized with 1% *I_2_-KI* solution in 4-day-old seedlings grown on 1/2 MS. Roots were stained for 1 minute, rinsed with water, cleared with 50% chloral hydrate for 45 seconds and photographed with Leica MZ95.

For the resin section, the 3 mm-length of root caps were obtained from plants grown on MS culture medium for 4 days. They were vacuum infiltrated for 1 h in 2.5% glutaral-dehyde, in 0.05 M phosphate buffer, pH 7.4 at room temperature and then in 4 °C overnight. Samples were then subsequently dehydrated in a graded acetone series at room temperature and embedded in 812 resin. Blocks were polymerized at 70°C for 24 hours, and cut into 1 μm sections on a RM2265 microtome (Leica, Heidelberg, Germany). For amyloplast staining, slides with sections attached were immersed in 0.5% periodic acid solution for 10 min, rinsed with distilled water, and then immersed in Schiff’s reagent (0.5% aniline red, 0.01M HCl, 1% sodium metabisulfite) for 15 min. then slides were rinsed, dried mounted by neutral balsam and observed microscopically.

### Starch Extraction and Quantification

Four-day-old seedlings with radicals approximately 6 cm in length were subjected to starch analysis. Roots cap segments were excised about 1 cm and pooled into samples from 10 plants each. Starch was extracted with 0.7 M perchloric acid and the insoluble fraction was cleared with 80% (v/v) ethanol three times then resuspended in water as described [34]. Samples were boiled for 10 min then starch was measured using the Starch (GO/P) Assay Kit(sigma)according to the manufacturer’s instructions.

### Salt Stress Treatment

WT and *OsGA2ox5-ox* rice seeds were incubated in water for two days, followed by incubation in water supplemented with 100 mM or 140 mM NaCl for 1 week. For *Arabidopsis*, Col-0 and *OsGA2ox5-ox* transgenic seeds were planted in Petri dishes containing solidified 1/2 MS medium and grown for two weeks. The seedlings were then transferred to 1/2 MS medium containing 170 mM NaCl; the seedlings were photographed three weeks later.

## Results

### *OsGA2ox5* is Widely Expressed in Various Rice Tissues

**Figure 1:**
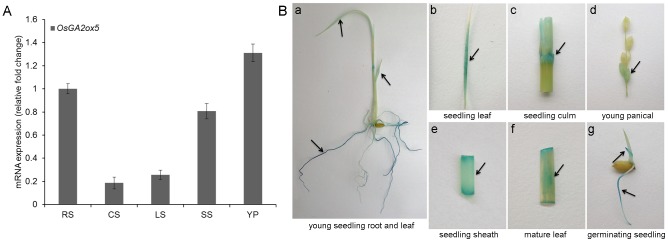
Expression pattern of *OsGA2ox5 in vivo*. **(A)** Real-time PCR analysis of *OsGA2ox5* in various organs of wild-type plants. RS, seedling root; CS, seedling culm; LS, seedling leaf; SS, seeding sheath; YP, young panical. The expression is relative to that of *OsActin*. Values are expressed as the average ± SD of three technical replicates, and the amount of *OsGA2ox5* in roots was set at 1.0; (B) Histochemical analysis of *P_OsGA2ox5_:GUS* gene activities in different tissues and organs of rice. The promoter region of *OsGA2ox5*, 3,500-bp upstream of ATG (*P_OsGA2ox5_*) was inserted upstream of the *GUS* gene at the *Xba I*-*Sma I* sites of the p1300GN-GUS vector. Arrows indicated the expression tissues of *OsGA2ox5*.

To determine the expression pattern of *OsGA2ox5* in rice, we analyzed *OsGA2ox5* expression in rice plants by real-time PCR using *OsGA2ox5*-specific primers. Rice *OsGA2ox5* was detected in the root, leaf, culm, sheath and young panicles of rice seedlings (Fig. 1A). To confirm the expression pattern of *OsGA2ox5*, an expression vector containing GUS (ß-glucuronidase) driven by the *OsGA2ox5* promoter was constructed and transformed into Zhonghua 11 (*Oryza sativa* L. subsp. *japonica*). Consistent with the results of real-time PCR assays, the transgenic plants showed GUS staining in the roots, culms, leaves, sheaths and panicles (Fig. 1B). These tissues always get staining at positions of a cut and suggest that this is mechanical.

### OsGA2ox5 is Localized to the Cytoplasm and Nucleus

**Figure 2:**
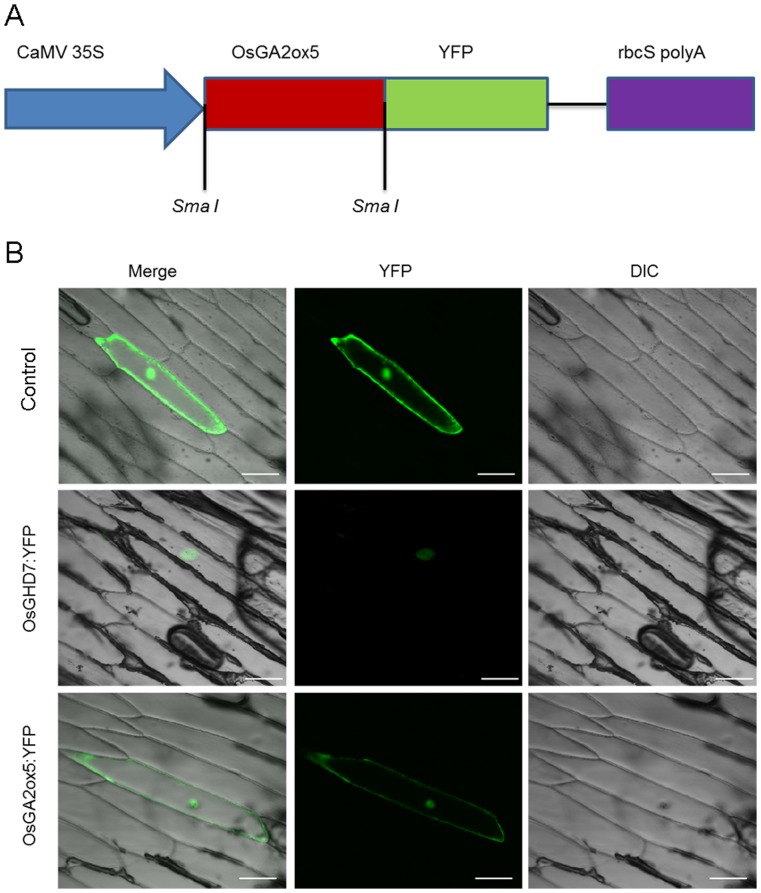
Localization of OsGA2ox5-YFP protein. (A) Diagram of the inserted region of the vector pA7:: OsGA2ox5::YFP; (B) Subcellular localization of OsGA2ox5. OsGA2ox5 was detected both in the cytoplasm and nucleus, the nucleus marker protein OsGHD7 was detected exclusively in the nucleus of onion epidermal cells and the control YFP showing signal both in cytoplasm and nucleus. DIC (Differential Interference Contrast), referring to bright field images of the cells.

To determine the subcellular localization of the OsGA2ox5 protein, The *OsGA2ox5* coding sequence was fused in frame to the N-terminus of YFP (Fig. 2A). The *OsGHD7* coding sequence was also fused in frame to the N-terminus of YFP under the control of the CaMV 35S promoter. The subcellular localization of the OsGA2ox5-YFP was examined through a transient expression of OsGA2ox5-YFP in onion epidermal cells. An examination of yellow florescence by confocal laser-scanning microscopy showed that YFP alone localized at the nucleus and cytosol of onion epidermal cells and the yellow fluorescent signal of OsGHD7 was detected exclusively in the nucleus of the onion epidermal cells, while OsGA2ox5-YFP was localized to the same region as YFP alone, i.e., the cytoplasm and nucleus (Fig. 2B). More than 30 YFP positive cells were detected. OsGA2ox5-YFP exhibited cytoplasm and nucleus localization in all these cells. These results demonstrated that OsGA2ox5 is a cytoplasm- and nuclear-localized protein.

### Overexpression of *OsGA2ox5* Produces a Severe Dwarf Phenotype

**Figure 3:**
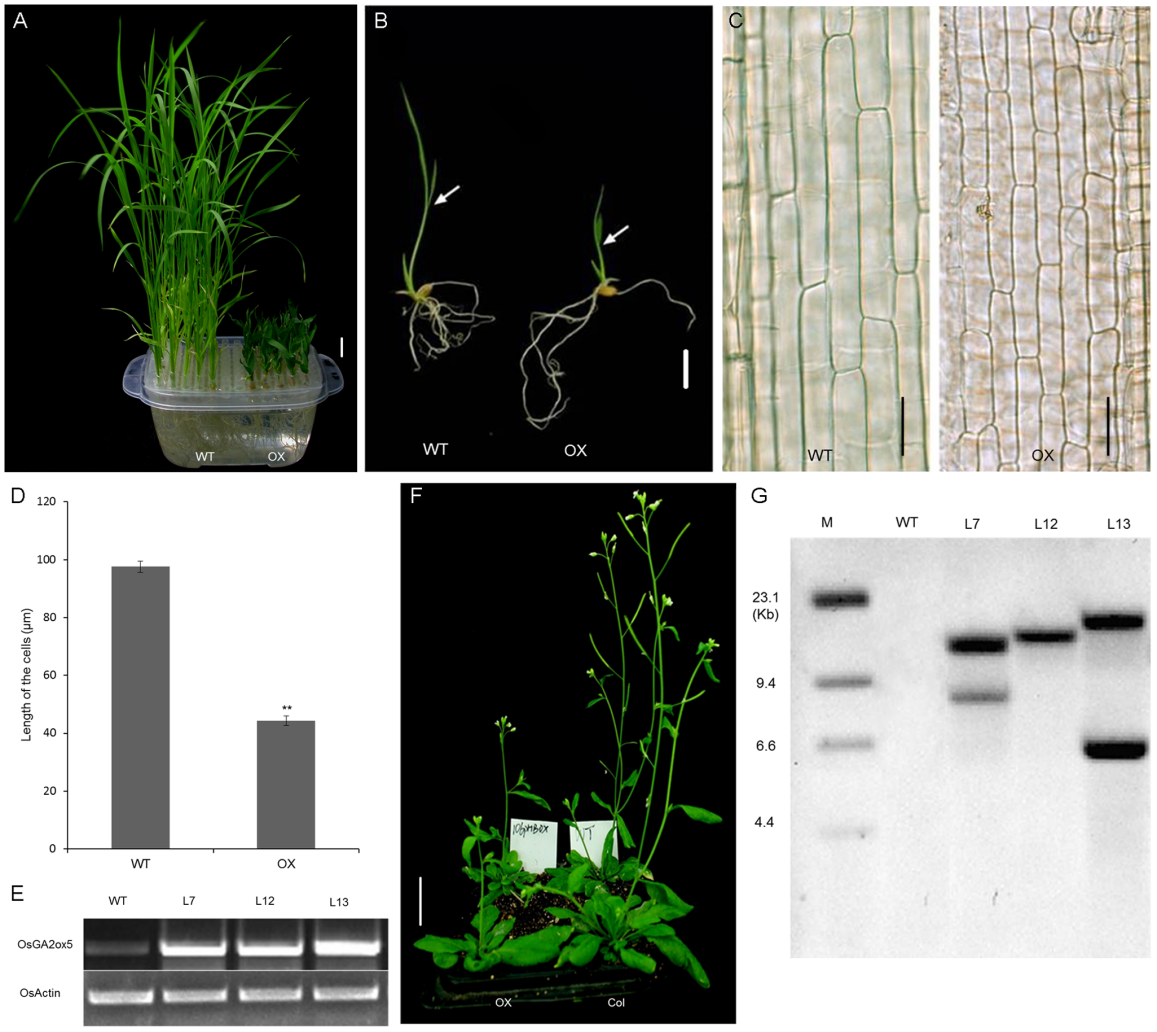
Phenotype of *OsGA2ox5*-overexpressing plants. (A) Phenotype of WT (left) and dwarf *OsGA2ox5-ox* plants (right). 2-week-old water cultured seedlings were used for photograph; (B) Arrows indicate the boundary between the second leaf sheath and the blade of 5-day-old water cultured seedlings. Bar = 1 cm; (C) Longitudinal sections of the elongated regions of the second leaf sheath of WT (left) and *OsGA2ox5-ox* plants (right). Bar = 25 µm; (D) Quantitative measurement of the cell length of second leaf sheath in WT and OX (n = 20). Error bars show standard errors (SE). Asterisks indicated significant differences at P <0.01 compared with the wild type by Student’s t test; (E) Ectopic expression of *OsGA2ox5* in *Arabidopsis.* Left is *OsGA2ox5* transgenic plants and wide type *Arabidopsis* (Col) is on the right. Plants photographed are 4-weeks-old. Bar = 2.5 cm; (F) Expression level of *OsOsGA2ox5* in transgenic rice; WT was used as a control; (G) Southern blotting analysis of transgenic plants. Restriction endonuclease *Hind III* was used to digest the genomic DNA from the leaf tissue. M, molecular marker; WT, wild type; L7, L12, L13, three transgenic lines.

To analyze the roles of *OsGA2ox5* in plants, *OsGA2ox5* was inserted into the pHB vector and overexpressed in *Arabidopsis* and rice under the control of the double CaMV 35S promoter. Transformants were selected based on hygromycin resistance. Stable inherited homozygous T3 plants of three independent transgenic lines (L7, L12, and L13) were examined by Southern blot analysis (Fig. 3E). Southern blotting produced one band in L12, while two bands were observed in L7 and L13 and no band was observed in the WT. These results suggested L12 was single integration in the genome and L13, L17 were double integration in the genome of transgenic plants. But all of those transgenic lines showing similar phenotype which means that the transgenic plants phenotypes were caused by *OsGA2ox5*. Moreover, RT-PCR analysis revealed that *OsGA2ox5* was overexpressed in both transgenic rice and transgenic *Arabidopsis* plants (Fig. 3D). The *OsGA2ox5-ox* rice plants exhibited a severe dwarf phenotype (Fig. 3A), as previously reported [23]. There was no obvious difference in root length between two-week-old WT and *OsGA2ox5-ox* plants, but the height of the transgenic plants was 75% lower than that of the WT. Longitudinal section analysis of the second leaf sheaths revealed that the cells of the *OsGA2ox5-ox* plants were markedly shorter and smaller than those of the WT (Fig. 3B). The flowering and heading stages were delayed by approximately 20 days in the *OsGA2ox5-ox* plants, and the spike length was shorter, compared with those of the WT. Also, the seeds of *OsGA2ox5-ox* were small and irregularly shaped, light green and not well filled. Similar results also were observed in transgenic *Arabidopsis*, such as slow growth and late flowering compared with the WT (Fig. 3C).

### The Dwarf Phenotype of Transgenic Plants is Rescued by the Application of Exogenous GA_3_

**Figure 4:**
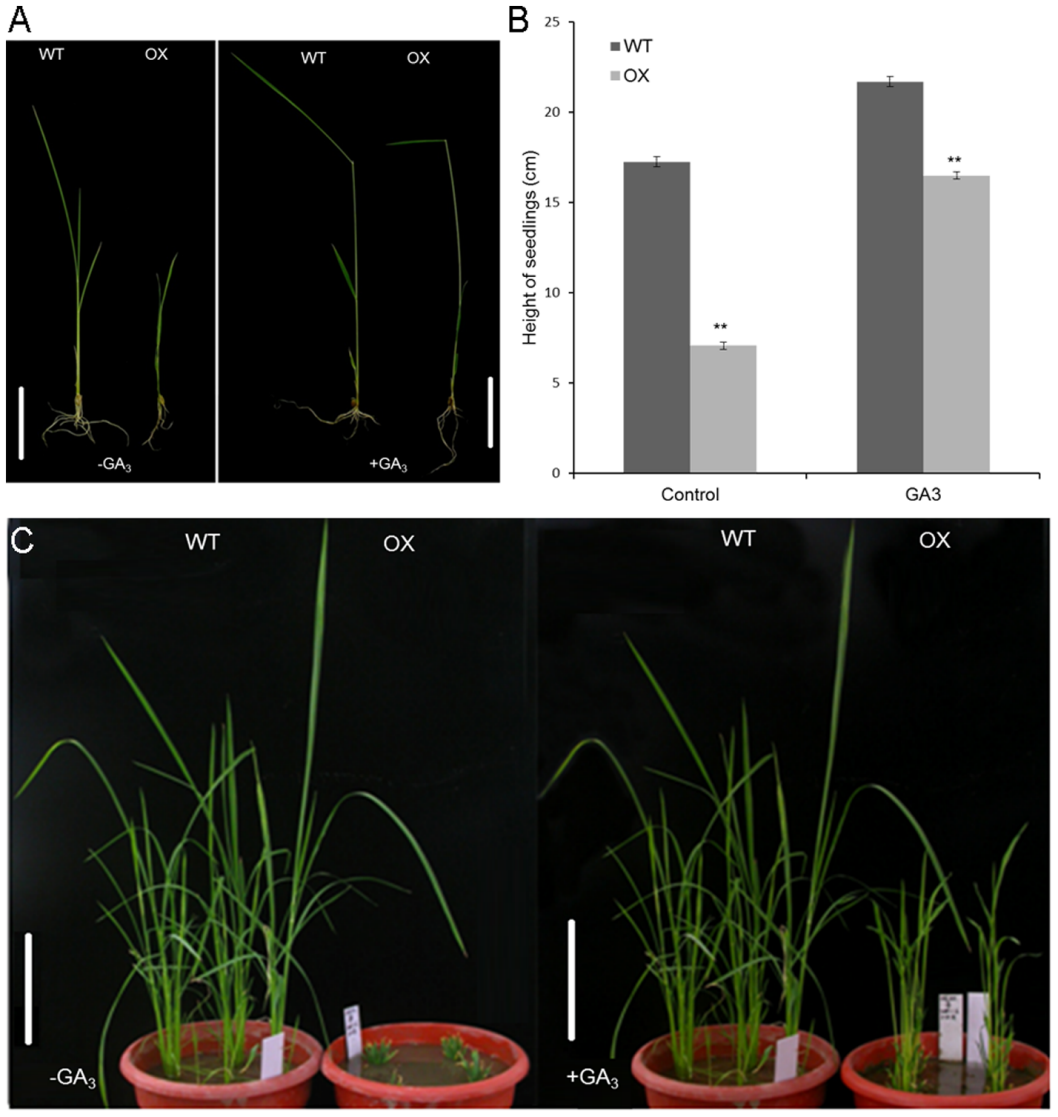
Exogenous GA3 effectively reverses the GA-deficiency phenotype. (A) Response to the application of GA_3_ in the plants. Two-week-old plants cultivated in MS liquid medium containing 1 µM GA_3_ or no GA_3_ for 1 week. Bar = 2 cm; (B) Plant elongation of *OsGA2ox5-ox* and WT seedlings treated with GA_3._ Plant height was measured at day 7 after GA_3_ treatment. Results represent three independent experiments with similar results. Error bars show standard errors (SE). Asterisks indicate significant difference at P <0.01 compared with the wild type by Student’s t test; (C) 1-month-old *OsGA2ox5-ox* and WT plants grown in a greenhouse and sprayed with exogenous GA_3_. Bar = 10 cm.

According to homology analysis result (Fig. S1) and previous data, we deduced that OsGA2ox5 can degrade C_20_-GA precursors. Overexpression of *OsGA2ox5* produced a severe dwarf phenotype, dark-green leaves and late flowering, which are all typical of GA-deficiency mutants. To investigate the responsiveness of *OsGA2ox5-ox* and WT plants to exogenous bioactive gibberellin, GA_3_, we cultivated WT and *OsGA2ox5-ox* plants in MS liquid medium containing 1 µM GA_3_ for 7 days. GA_3_ partially restored the height of *OsGA2ox5-ox* plants (Fig. 4A and B). Plants grown in a greenhouse and sprayed with exogenous GA_3_ exhibited a similar phenotype (Fig. 4C); the spike length, grain number and 1,000-grain weight were higher in plants sprayed with exogenous GA_3_ than in the control (Figure S2).

### GA Biosynthesis and Signaling is Regulated by *OsGA2ox5* Expression

**Figure 5:**
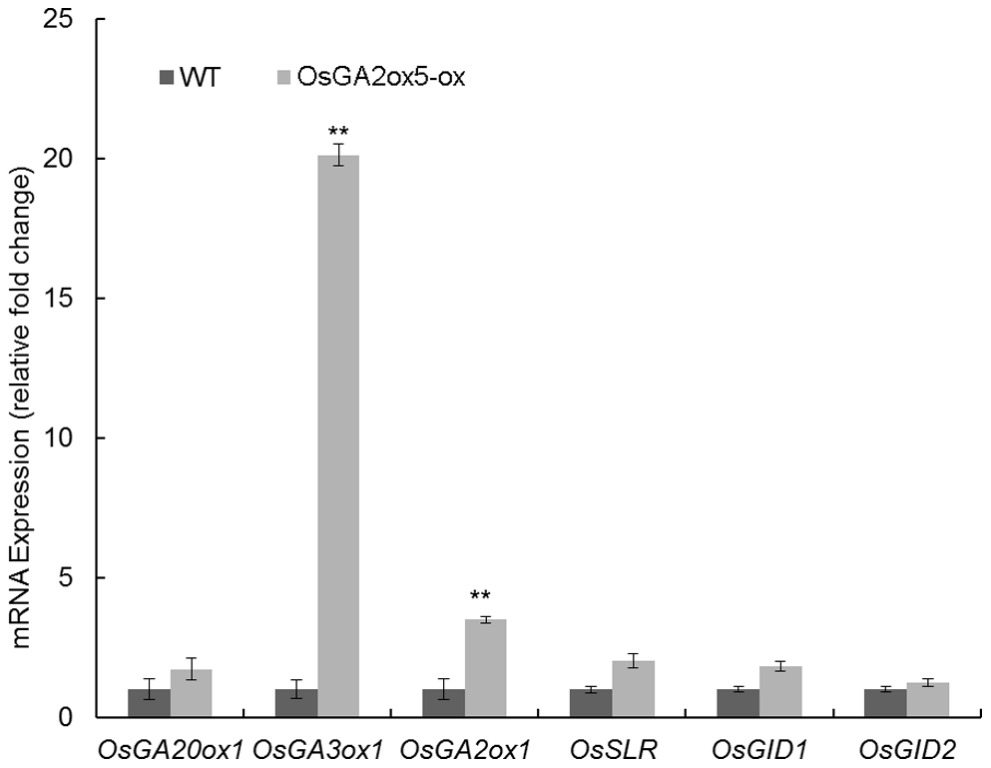
Gene expression analyses. The transcription levels of gibberellin metabolism and signal pathway genes expression levels analyzed by Real-time PCR. The rice *OsActin* gene was used as an internal control. Data are the mean ±SE of three independent measurements with three repeats. Asterisks indicate significant difference at P <0.01 compared with the wild type by Student’s t test. The expression of all of these GA biosynthesis and GA signaling genes was up-regulated in OX plants, especially the *OsGA3ox1* gene nearly 10-fold up-regulated. 3-week-old rice plants cultivated in water were used for experiments. *OsGA20ox1* and *OsGA3ox1* encode GA biosynthesis enzymes; *OsGA2ox1* encodes an enzyme that functions in GA degradation; *OsSLR* encodes a negative GA regulator in GA signaling and *OsGID1* and *OsGID2* encode GA receptors in rice.

OsGA2ox5 acts on C_20_-GA precursors, resulting in reduced levels of bioactive GA synthesis *in vivo*. In rice, the overexpression of *OsEUI* and *OsGA2ox6* altered the expression of GA signaling genes, and mutations in *AtGA2ox7* and *AtGA2ox8* resulted in the down regulated expression of GA_5_ [25], [26], [35]. To investigate whether overexpression of *OsGA2ox5* also regulates the expression of GA biosynthesis and GA signaling genes, we used real-time PCR assays to detect the expression of genes encoding GA20oxidase, GA3oxidase and GA2oxidase [36], [37] as well as *OsSLR* [16] and *OsGIDs* [17], [38]–[40]. The expression of all of these GA biosynthesis genes was up-regulated in *OsGA2ox5-ox* plants, especially the *OsGA3ox1* gene, which encodes the enzyme that catalyzes the last step of GA synthesis (Fig. 5). Interestingly, the GA catalysis gene *OsGA2ox1,* GA signaling genes, the receptor gene *OsGID1*, the F-box gene *OsGID2* and the gene encoding rice DELLA protein OsSLR, a negative GA regulator, were all up-regulated in *OsGA2ox5-ox* plants compared with the WT.

### *OsGA2ox5* Affects Root Starch Granule Development and Gravitropism

**Figure 6:**
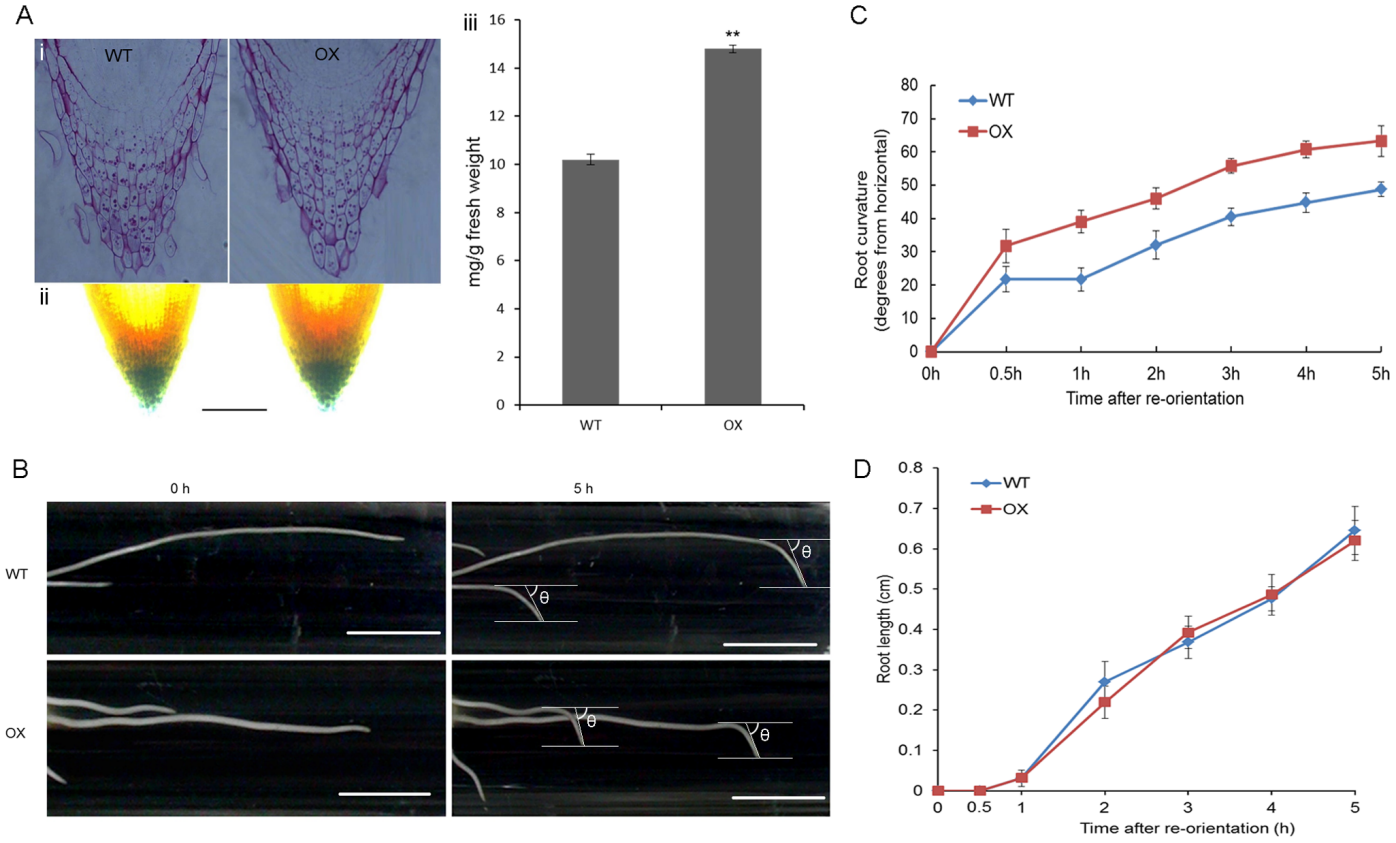
*OsGA2ox5* affects root starch granule development and gravitropism. (A) Starch staining and detection of wild-type and *OsGA2ox5-ox* plants root caps. i, Resin section of WT and OX plants root caps and ii, *I_2_-KI* staining of the WT and OX plants root caps. Figures showing that OX plants have increased cell layers in the enlarged root caps compared to WT; iii, starch content in root caps of WT and OX plants. Roots cap segments were excised about 1 cm and pooled into samples from 10 plants each for experiments. Results represent three independent experiments with similar results. Bar = 100µm. Asterisks indicate significant difference at P <0.01 compared with the wild type by Student’s t test; (B) Gravity response of light-grown 4-day-old WT and OX seedling roots. After reorientation the OX seedling roots bent faster than WT. θ indicated the roots bending angle of the WT and OX plants respectively after reorientation at 5h. Experiments were performed three times with similar results. Bar = 1 cm; (C) Time course of root gravitropical curvature (after reorientation). Light-grown wild-type and *OsGA2ox5-ox* seedlings were displaced by 90° and monitored for the orientation of the primary root caps. The vertical position is represented by 90°, and the horizontal position is represented by 0°. Data shown are the means ± SE of 30 seedlings; (D) Time course of root length (after reorientation). Data shown are the means ± SE of 20 seedlings.

A previous study revealed that GA plus kinetin causes the complete destarching of amyloplasts [41]. In addition, the GA-degrading enzyme OsEUI also alters rice root granule development and gravity responses [42]. We want to know whether *OsGA2ox5* had a similar effect on root starch granule development and gravitropism in rice. By extracting and quantifying the root cap starch, we found *OsGA2ox5-ox* plants generated more starch granules (14.9 mg/g) than the WT plants (10.2 g/mg). Then we using two methods to staining the root caps starch granule and depend on more than 20 staining results we found out that those increased starch granules in *OsGA2ox5-ox* plants were caused by increased cell layers in the enlarged root caps (Fig. 6A). Then, we analyzed the gravitropic response of WT and *OsGA2ox5-ox* roots in light-grown seedlings. *OsGA2ox5-ox* roots bent more quickly than the WT in response to gravity. The roots of *OsGA2ox5-ox* seedlings exhibited an accelerated gravity response; most *OsGA2ox5-ox* root caps were nearly vertical 5 h after rotation (Fig. 6B and C). And the roots growth rates showed no significant difference between WT and OX after reorientation (Fig. 6D), suggesting the quick bending of *OsGA2ox5-ox* roots than the WT in response to gravity was not due to the different root growth rates between them.

### Transgenic Plants Overexpressing *OsGA2ox5* Showed Increased Tolerance to High Salinity Stress

**Figure 7:**
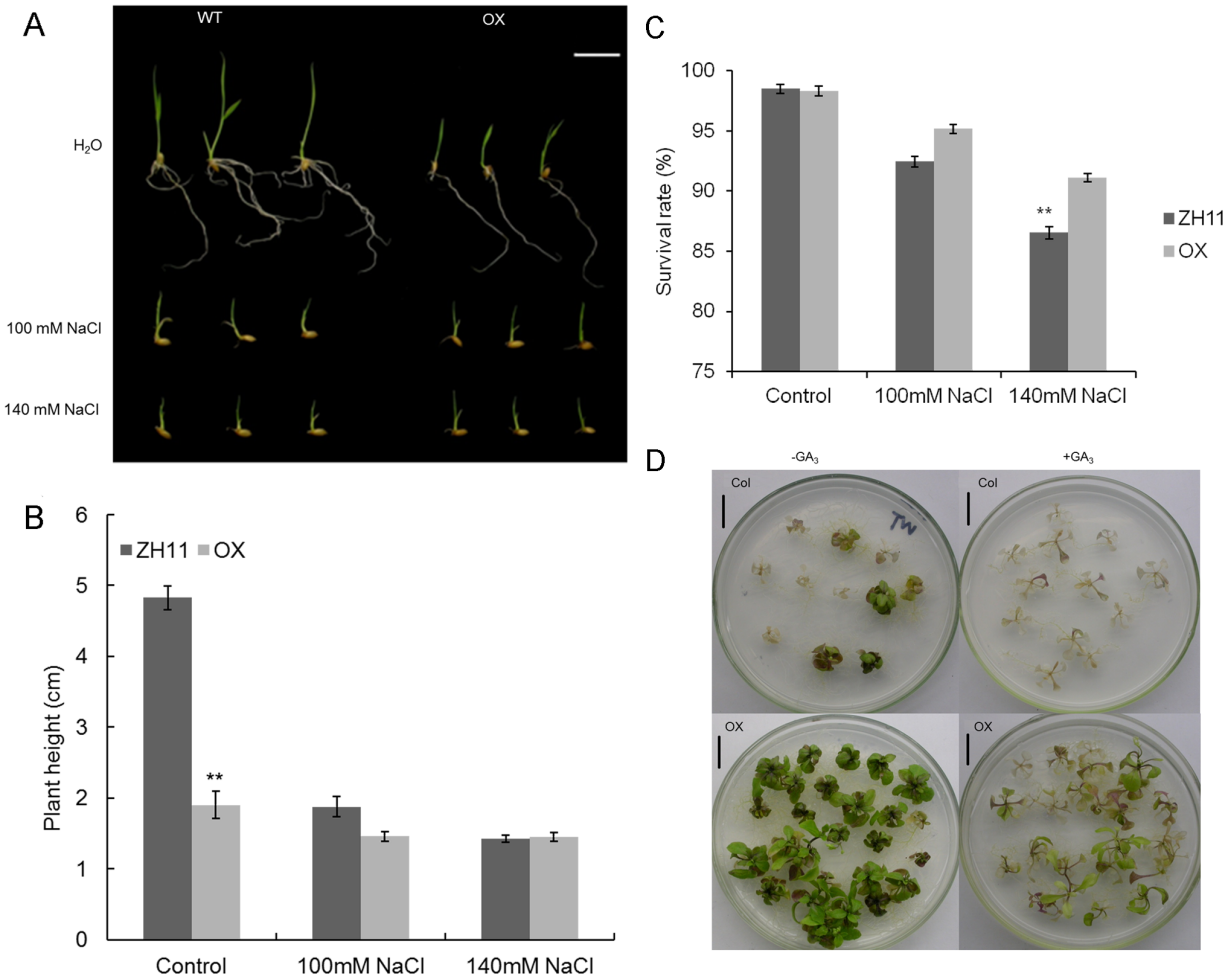
Effects of salinity stress on plant growth. (A) Phenotype of WT and OX rice seedlings under salt treatment. Photographs were taken after 5 d of growth in water (control) and 100 mM or 140 mM NaCl. For each treatment, 20 seedlings were measured. Bar = 1 cm; (B) Statistics analysis of plants height under salt treatment. WT and OX plants stem height at 5 days of growth in various concentrations of NaCl. Bar = 1 cm. Asterisks indicate significant difference at P <0.01 compared with the wild type by Student’s t test; (C) Quantitative analysis of survival rates under salt treatment. The results are averages of three independent experiments with 30 plants per experiment. Asterisks indicate significant difference at P <0.01 compared with the wild type by Student’s t test; (D) Phenotype of wide type *Arabidopsis* (Col) and transgenic *Arabidopsis* with and without GA_3_ under salt treatment. Physiological changes in WT and *OsGA2ox5-ox* transgenic *Arabidopsis* plants transferred to plates containing 170 mM NaCl. Photographs were taken 3 weeks after transfer. +GA represents treatment with 10 µM GA_3_.

A previous study showed that *AtGA2ox7* was directly activated by a transcription factor of the *DREB1/CBF* subfamily DDF1, and showed resistance to high salinity [43]. OsGA2ox5 shares high homology with AtGA2ox7 and may therefore also be responsive to salt stress. To test this, *OsGA2ox5-ox* transgenic rice plants that were geminated in water were transferred to water containing 100 mM or 140 mM sodium chloride (Fig. 7A). As shown, on day 7 after transfer, high salinity restricted plant growth, compared with plants grown in water. The height of the WT plants under 100 mM and 140 mM sodium chloride conditions were reduced by 53% and 60%, respectively, compared with water-grown plants, while the *OsGA2ox5-ox* plants exhibited only a 25% reduction in either 100 mM or 140 mM sodium chloride compared with the control (Fig. 7B). Consistently, more than 95% and 91% of the *OsGA2ox5-ox* plants survived under 100 mM and 140 mM sodium chloride conditions, whereas 92% and 86% of the Zhonghua 11 plants survived (Fig.7C). To clarify the role of *OsGA2ox5* during high salinity stress, we also examined the growth of transgenic *OsGA2ox5 Arabidopsis* under high-salt conditions (Fig. 7D). Transgenic plants grown on 1/2 MS medium for 2 weeks were transferred to 1/2 MS medium containing 170 mM NaCl. After 21 days, most WT plants died, however, the survival rate of *OsGA2ox5-ox* plants was very high. When 1/2 MS medium containing 170 mM NaCl was supplied with 10 µM GA3, both WT and *OsGA2ox5-ox* plants showed reduced survival rates. These results demonstrate that GA reduces salinity tolerance, and *OsGA2ox5* is related to salt-stress tolerance.

## Discussion

The plant hormone GA is very important; GA homeostasis is essential for normal plant growth and development as well as environmental adaptation. In plants, the level of bioactive GAs is accurately maintained by the regulation of GA biosynthesis and catabolism. To date, two types of enzymes are known to regulate GA biosynthesis and catabolism. GA2oxs and OsEUI [21], [23], [27] act on bioactive GAs and their precursors to reduce the level of bioactive GAs, thereby maintaining GA homeostasis. GA2oxs catalyze the catabolism of bioactive GAs and GA precursors into inactive GAs by hydroxylating the C_2_ of C_19_-GAs and C_20_-GAs [21]. EUI P450 converts bioactive GA_4_ and its precursor GA_9_ into inactive 16, 17 epoxy-GAs by 16, 17-epoxidation [26], [27]. In this study, we cloned *OsGA2ox5* from *Oryza sativa*. Real-Time PCR assays and GUS staining revealed that this gene is expressed in roots, culms, leaves, sheaths and panicles (Fig. 1A and B). The high level of *OsGA2ox5*-GUS expression in culm suggests that *OsGA2ox5* functions in plant elongation. OsGA2ox5 is localized to both the nucleus and cytoplasm (Fig. 2), which is similar to the localization of OsGA2ox6 [25]. Previous studies have suggested that OsGA2ox5 only hydroxylates C_20_-GA substrates in rice, and *OsGA2ox5* belongs to the subgroup C_20_GA2ox, which also contains *AtGA2ox7*, *AtGA2ox8*, *SoGA2ox3 OsGA2ox6* and *OsGA2ox9* [21], [23], [35]. Amino acid sequence alignment showed that OsGA2ox5 is similar to AtGA2ox8 and SoGA2ox3 and has the three unique conserved motifs (Fig. S1) [21]. Therefore, we deduced that OsGA2ox5 can act on C_20_-GA substrates to produce inactive GAs.

Overexpression *OsGA2ox5* in rice and *Arabidopsis* produced plants that were dwarfed and dark green with retarded growth (Fig. 3A and C). The dwarf phenotype of *OsGA2ox5*–overexpressing rice plants was restored by exogenous GA_3_ treatment (Fig. 4A and C). This suggests that the overexpression of *OsGA2ox5* decreases the level of bioactive GAs in rice, and the dwarf phenotype is caused by a shortage of bioactive Gas; these plants exhibit the typical GA-deficiency phenotype. Similar to the overexpression of *AtGA2ox7* and *AtGA2ox8* in *Arabidopsis*, and *OsGA2ox6* in rice, *OsGA2ox5-ox* plants exhibited the dwarf phenotype [25], [35].

We then examined the expression of genes encoding GA biosynthesis and metabolism enzymes, as well as GA signaling pathway genes (Fig. 5). The GA biosynthesis genes *OsGA20ox1* and *OsGA3ox1* were up-regulated in *OsGA2ox5-ox* plants compared with WT. The expression of *OsGA20ox1* was slightly increased in *OsGA2ox5-ox* plants, while the expression of *OsGA3ox1*, which encodes the last key enzyme in the synthesis of bioactive GAs, was increased sharply (nearly 10-fold) in *OsGA2ox5-ox* plants. This may represent a type of feedback regulation required for plants to maintain a stable endogenous GA level, as the overexpression of *OsGA2ox5* decreased the endogenous GA level, and plants must synthesize increasing amounts of bioactive GAs to maintain GA homeostasis. Therefore, GA synthesis genes were up-regulated in the *OsGA2ox5-ox* plants, especially *OsGA3ox1*. Consistent with this notion, the upregulation of *GA20ox* and *GA3ox* has been reported in GA-deficient and GA-insensitive mutants [44], [45]. We also examined the expression of *OsGA2ox1*, which is involved in GA catabolism. The expression of *OsGA2ox1* was also increased in *OsGA2ox5-ox* plants, which was perhaps influenced by the increased expression of *OsGA3ox1*. Interestingly, the expression of GA signaling genes that were examined was up-regulated in the *OsGA2ox5-ox* plants. The expression of *GID1* and *GID2*, which encode receptors of GA and the rice DELLA protein SLR, was also increased in *OsGA2ox5-ox* plants. Bioactive GAs interact with SLR by binding to its receptors (GID1, GID2), thereby decreasing the activity of SLR protein in GA signal transduction [17], [40]. Perhaps a similar mechanism occurs during the feedback regulation of GA biosynthesis, which is also influenced by altered levels of bioactive GAs. Decreased levels of bioactive GAs increase the expression level of *GID1* and *GID1*, and this feedback stimulates the expression of *SLR*. This phenomenon was also identified in *EUI-ox* plants [42].

In addition, the root caps of *OsGA2ox5-ox* rice plants exhibited a stronger gravitropic response than WT plants (Fig. 6B and C), and not caused due to the growth speed (Fig. 6D), along with more starch granules (Fig. 6A). *EUI-ox* plants also have more starch granules than WT [42]. GA induces amylase activity, which can degrade starch [46]. Perhaps the increased gravitropism in *OsGA2ox5-ox* plants is due to altered levels of bioactive GAs.

We also found that the *OsGA2ox5-ox* plants showed increased stress tolerance. *OsGA2ox5-ox* seedlings grown in water containing 100 or 140 mM NaCl showed little decrease in growth (vs. water-grown seedlings) compared with WT rice plants grown under high salinity conditions (Fig. 7A). *Arabidopsis* plants ectopically expressing *OsGA2ox5* also exhibited salinity resistance (Fig. 7C), which is consistent with the phenotype of *AtGA2ox7-ox Arabidopsis* plants [43]. Overexpression of the *DWARF AND DELAYED FLOWERING 1* (*DDF1*) gene, which encodes an *AP2* transcription factor of the *DREB1/CBF* subfamily, causes dwarfism and salinity tolerance. The *AtGA2ox7* gene is strongly up-regulated in *DDF1*-overexpressing transgenic *Arabidopsis*, and its promoter has a DDF1 binding motif. This suggests that *AtGA2ox7* is a direct target of the *DDF1* transcriptional activator [47]. Transgenic *AtGA2ox7-ox Arabidopsis* also exhibits the salinity tolerance phenotype [43]. According to sequence analysis, some *DREB1/CBF* binding motifs are present in the promoter of *OsGA2ox5* [48]. Therefore, perhaps the salinity tolerance of *OsGA2ox5-ox* transgenic plants is due, at least in part, to the influence of *DREB1/CBF*.

In summary, we demonstrated that the overexpression of *OsGA2ox5* in plants produces a dwarf phenotype, and this phenotype is restored by exogenous GA_3_ treatment. The overexpression of *OsGA2ox5* altered the expression levels of GA biosynthesis and GA signaling genes, leading to a series of responses, including increased stress resistance and increased gravitropism.

## Supporting Information

Figure S1 Comparison of the deduced amino acid sequences of OsGA2ox5 with other GA2-oxidases. (A) Amino acid sequence alignment of rice GA2oxs (OsGA2ox1, OsGA2ox5, OsGA2ox6 and OsGA2ox9), Arabidopsis GA2oxs (AtGA2ox1, AtGA2ox7 and AtGA2ox8) and spinach GA2ox (SoGA2ox3) using the DNAMAN software. C_20_ GA2oxs (OsGA2ox5, OsGA2ox6, OsGA2ox9, AtGA2ox7, AtGA2ox8, and SoGA2ox3) contain three highly conserved sequence motifs (underlined with Roman numerals) that are absent in all C_19_ GA2oxs (OsGA2ox1 and OsGA2ox3 as examples for comparison). (B) Phylogenetic analysis of these GA2-oxidase proteins.
(TIF)

Figure S2 1000-grain weight of main panicle (A), grains number of each spike (B), Height of stem (C), Length of spike (D) of transgenic lines overexpressing *OsGA2ox5* and wild type Zhonghua 11 under normal and GA_3_ condition. Twelve samples were measured for plant height, spike length and grains number of each line. 1,000-seed weight was measured in triplicate.
(TIF)

Table S1 Primers.
(XLS)
