# Supplementary figures and images for: OsGA2ox5, a Gibberellin Metabolism Enzyme, Is Involved in Plant Growth, the Root Gravity Response and Salt Stress
Source: PLoS One. 2014 Jan 27;9(1):e87110. doi: 10.1371/journal.pone.0087110 (PMC3903634; doi:10.1371/journal.pone.0087110)

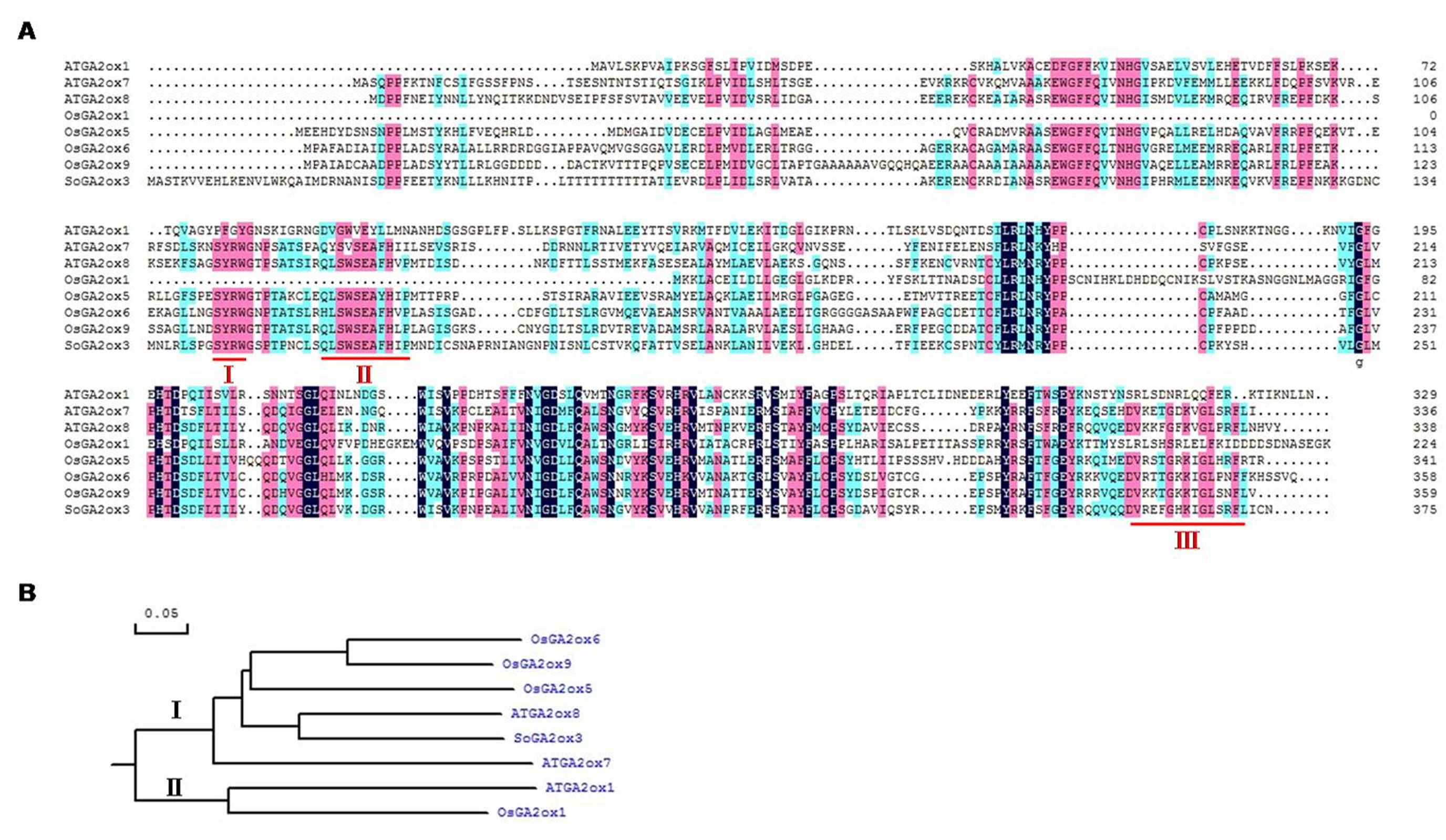

Supplement: Figure S1 — Comparison of the deduced amino acid sequences of OsGA2ox5 with other GA2-oxidases. (A) Amino acid sequence alignment of rice GA2oxs (OsGA2ox1, OsGA2ox5, OsGA2ox6 and OsGA2ox9), Arabidopsis GA2oxs (AtGA2ox1, AtGA2ox7 and AtGA2ox8) and spinach GA2ox (SoGA2ox3) using the DNAMAN software. C20 GA2oxs (OsGA2ox5, OsGA2ox6, OsGA2ox9, AtGA2ox7, AtGA2ox8, and SoGA2ox3) contain three highly conserved sequence motifs (underlined with Roman numerals) that are absent in all C19 GA2oxs (OsGA2ox1 and OsGA2ox3 as examples for comparison). (B) Phylogenetic analysis of these GA2-oxidase proteins. (TIF) [file pone.0087110.s001.tif]

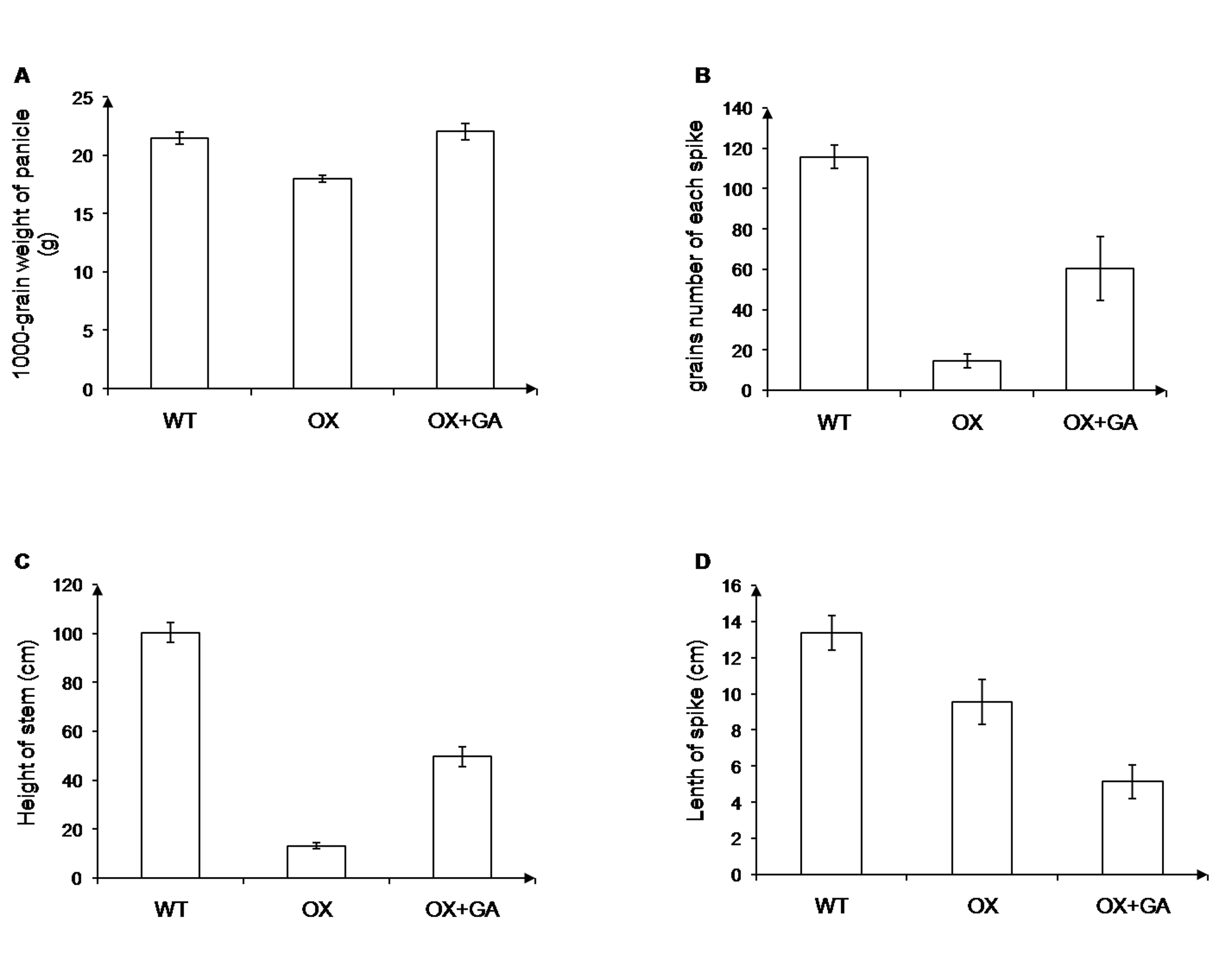

Supplement: Figure S2 — 1000-grain weight of main panicle (A), grains number of each spike (B), Height of stem (C), Length of spike (D) of transgenic lines overexpressing OsGA2ox5 and wild type Zhonghua 11 under normal and GA3 condition. Twelve samples were measured for plant height, spike length and grains number of each line. 1,000-seed weight was measured in triplicate. (TIF) [file pone.0087110.s002.tif]
